# Sensorineural hearing loss after concurrent chemoradiotherapy in nasopharyngeal cancer patients

**DOI:** 10.1186/1748-717X-6-19

**Published:** 2011-02-20

**Authors:** Janjira Petsuksiri, Achariyaporn Sermsree, Kullathorn Thephamongkhol, Phawin Keskool, Kanthong Thongyai, Yaowalak Chansilpa, Pittayapoom Pattaranutaporn

**Affiliations:** 1 Division of Radiation Oncology, Faculty of Medicine Siriraj Hospital, 2 Prannok Rd, Bangkoknoi, Bangkok, Thailand; 2 Department of Radiation Oncology, Ubonratchathani Cancer Center, Ubonratchathani, Thailand; 3 Department of Oto-Rhino-Laryngology, Faculty of Medicine Siriraj Hospital, 2 Prannok Rd, Bangkoknoi, Bangkok, Thailand

## Abstract

**Background:**

Sensorineural hearing loss (SNHL) is one of the major long term side effects from radiation therapy (RT) in nasopharyngeal cancer (NPC) patients. This study aims to review the incidences of SNHL when treating with different radiation techniques. The additional objective is to determine the relationship of the SNHL with the radiation doses delivered to the inner ear.

**Methods:**

A retrospective cohort study of 134 individual ears from 68 NPC patients, treated with conventional RT and IMRT in combination with chemotherapy from 2004-2008 was performed. Dosimetric data of the cochlea were analyzed. Significant SNHL was defined as > 15 dB increase in bone conduction threshold at 4 kHz and PTA (pure tone average of 0.5, 1, 2 kHz). Relative risk (RR) was used to determine the associated factors with the hearing threshold changes at 4 kHz and PTA.

**Results:**

Median audiological follow up time was 14 months. The incidence of high frequency (4 kHz) SNHL was 44% for the whole group (48.75% in the conventional RT, 37% with IMRT). Internal auditory canal mean dose of > 50 Gy had shown a trend to increase the risk of high frequency SNHL (RR 2.02 with 95% CI 1.01-4.03, p = 0.047).

**Conclusion:**

IMRT and radiation dose limitation to the inner ear appeared to decrease SNHL.

## Background

Radiation therapy (RT) is the standard treatment for nasopharyngeal cancer (NPC) patients as a result of the relative radiosensitivity, deep location and the close proximity to the normal critical structures. High dose RT of ≥ 66 Gy in combination with chemotherapy has yielded a 5-year locoregional control for more than 80% of the patients with locally advanced disease [1-3]. Consequently, RT produces undesirable side effects on the adjacent organs. In addition to xerostomia, sensorineural hearing loss (SNHL), resulting from the cochlea damage, is one of the major long term side effects which impacts the patients' quality of life. With modern conformal radiation techniques, the incidence of radiation induced SNHL is expected to decline, due to a better visualization of the organs on the planning CT images and a better capability to spare the cochlea with a mean dose < 40-50 Gy [4-7].

This retrospective analytic study aims to report the incidences of SNHL of NPC patients receiving chemoradiotherapy with conventional RT comparing with intensity modulated radiation therapy (IMRT). To our knowledge, this study is the first one to compare hearing status between conventional RT and IMRT for NPC patients. As most earlier studies had some disagreement about the cochlea contouring for dose volume analysis, the further aim of this study is to evaluate radiation doses in each specific part of the inner ear [cochlea, inner ear (cochlea and vestibule) and internal auditory canal (IAC)] in correlation with the incidences of SNHL.

## Methods

The medical records, including radiation dosimetric data and audiological assessment of the 507 NPC patients receiving definitive RT at the division of Radiation Oncology, Siriraj Hospital from January 2004 to December 2008 were retrospectively reviewed under the approval of the Siriraj institutional review board.

**Figure 1:**
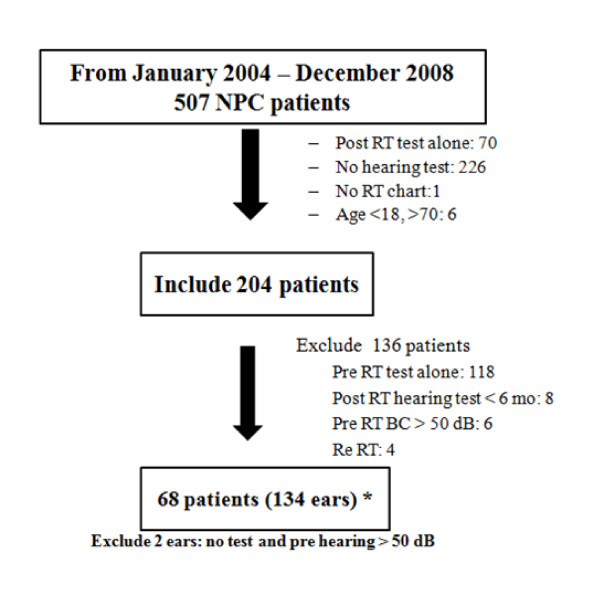
**Patients flow diagram**.

Two hundred and four NPC patients with T1-T4, N0-N3, M0 diseases (according to AJCC 1997 staging system) who completed RT courses with either conventional RT or IMRT with baseline pre RT audiograms were included. Patients were excluded from the study when they had no medical records, no post RT audiograms, or not completed RT. Patients who had tumor invasion into the inner ear or had a recurrent disease were also excluded. No patients were excluded because of a hearing impairment during RT. Patients who had severe hearing impairment (pure tone average: PTA, at 0.5, 1, 2 kHz > 50 dB in both ears) on pre RT audiograms were excluded. Each individual ear was evaluated independently for radiation doses and hearing status. Ultimately, 134 individual ears with intact hearing status were included for data analysis (Figure 1).

### Radiation therapy

The radiation technique has changed from conventional RT (before 2007) to IMRT (since 2007) due to machine evolution at our institute. After the start of the IMRT era, all patients with a curative aim were treated with IMRT. Therefore, 41 patients were treated with conventional RT and 27 patients were treated with IMRT. For the conventional RT, radiation was prescribed to a total dose of 66-70 Gy at 2 Gy per fraction, 5 fractions per week. All patients were treated with a Cobalt 60 teletherapy unit. Parallel opposed portals were used for the primary tumor site and the upper neck. Spinal cord and brainstem were mostly shielded at the dose of 46 Gy. This conventional field generally included the base of the skull, for which the inner ear was not intentionally protected by the posterior fossa block. The lower neck was routinely treated with the anterior split field.

For IMRT, the target volumes and normal tissue structures were defined by using CT images. The gross target volume (GTV) consisted of the gross primary tumor and involved lymph nodes as defined by contrast enhancement CT. Generally, clinical target volume (CTV) high risk was defined by adding a 5-mm margin to GTV. A smaller margin (3 mm) was accepted for the margin that was in close proximity to the critical structures, such as brainstem, optic nerves and optic chiasm. CTV intermediate and low risk regions were contoured according to the RTOG recommendation [8]. Planning target volume (PTV) was defined by adding a 5-mm margin to the CTVs in all dimensions to include setup uncertainties. Radiation doses were prescribed simultaneously to total doses of 66-70 Gy to the high risk region, 59.4-63 Gy to the intermediate-risk region, and 50.4-57 Gy to the low-risk region, in 33-35 fractions. The primary tumor and the upper neck were treated with IMRT. For the lower neck region, either continuing IMRT with the upper neck part or with the anterior spilt field was allowed.

### Dose Calculation of the Inner Ear

**Figure 2:**
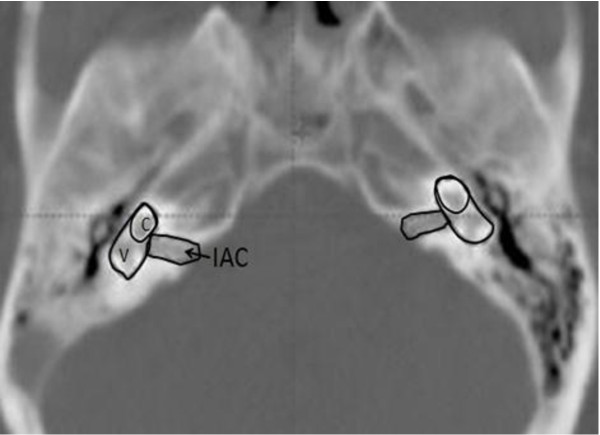
**Inner ear contouring**. C: Cochlea, V: Vestibule, IAC: internal auditory canal Inner ear = cochlea(C) + vestibule (V).

Dose calculation of the inner ear was not accessible for patients who received conventional RT. For the patients who were treated with IMRT, dose calculations to the inner ear were evaluated. Initially, the inner ears (cochlea and vestibule) were contoured and constrained (mean doses constraint of 35 Gy with doses accepted at 50 Gy) at the time of radiation treatment planning. Each of the inner ear structures was re-contoured (using bone window; window width = 2000 HU, window level = 400 HU) and reviewed by the authors (JP and AS) as in Figure 2. We defined the inner ear as a combination of the cochlea and vestibule. The purpose of the inner ear delineation was to compare its' dose with the prior studies that defined the inner ear as a cochlea for SNHL evaluation [6,7]. The IAC was contoured to evaluate the radiation doses to the cochlea nerve, which can be affected by radiation. The minimum dose, maximum dose and mean dose were recalculated for each part of the auditory pathway.

### Chemotherapy

Patients with locally advanced disease received concurrent intravenous platinum-based chemotherapy (Cisplatin or Carboplatin). Cisplatin was given with 100 mg/m^2 ^every 3 weeks during the radiation course, followed by Cisplatin 80 mg/m^2 ^at day 1 and 5-FU 1000 mg/m^2 ^at day 1-4 every 3 weeks. Carboplatin was allowed in patients with poor renal function or were intolerant to Cisplatin. Carboplatin was given in a weekly fashion (AUC 2) during the radiation course, followed by adjuvant Carboplatin (AUC 5 at day 1) in combination with 5-FU (1000 mg/m^2 ^at day 1-4) every 3 weeks. Type (Cisplatin or Carboplatin), doses, and cycles of chemotherapy were recorded.

### Audiological assessment

Pre and post RT audiological data were reviewed. The audiograms were ordered routinely for all patients at pre RT and post RT periods by ENT physicians per our hospital's policy. The bone conduction (BC) threshold was measured at 0.5-4 kHz to detect the early SNHL from the cochlea and/or IAC damages. BC threshold at 4 kHz was selected to represent the high frequency loss. The pure tone average (PTA), an average of threshold levels at 0.5 kHz, 1 kHz and 2 kHz, was chosen to reflect the threshold in the low frequency speech range [9,10]. Post RT audiograms (at least 6 months after completion of RT) were obtained at various intervals. The most recently performed audiograms were used for the analysis. Hearing threshold changes were determined by comparing with their pre RT baselines. As per the American Speech and Hearing Association guidelines, significant SNHL was defined as a ≥ 10 dB increase at two consecutive frequencies or ≥ 15 dB at one frequency. Hence, the cut-off point of ≥ 15 dB increase from baseline in BC threshold at 4 kHz was used as a criterion for SNHL in this study.

The incidences of otitis media effusion (OME) and tympanic membrane perforation were documented at baseline and follow up. Influences from age, chemotherapy, OME, co-morbidities (DM and hypertension), radiation techniques and the radiation doses on the change of BC thresholds were assessed.

### Statistical methods

The statistics program STATA, version 8 was employed for data analysis. Relative risk (RR) with 95% confidential interval (CI) was used to determine the relationship between the possible associated factors and the threshold changes at 4 kHz and PTA. We tested the null hypothesis as to whether the relative risk was equal to 1 by calculating the chi-square test statistics.

## Results

From January 2004 to December 2008, 68 patients (41 patients with conventional RT, 27 patients with IMRT) were enrolled for the hearing analysis. The patients' characteristics were shown in Table 1. Sixty six patients (97.1%) received concurrent chemoradiotherapy and only 2 patients (2.9%) received RT alone.

**Table 1:** Patient characteristics (Total 134 individual ears, 68 patients)

Characteristics	Value (68 patients)
**Age **(18-70 years old)	Median 47.5 years old
≤ 50	44 (65%)
> 50	24 (35%)
**Co-morbidities (patients)**	
DM and/or Hypertension	14 (20.6%)
No co-morbidities	54 (79.4%)
**Pre RT otitis media (ears)**	
Yes	34 (25.4%)
No	71 (53%)
No data	29 (21.6%)
**Post RT otitis media (ears)**	
Yes	31 (23.1%)
No	93 (69.4%)
No data	10 (7.5%)
**Tumor stage**	
**I/II**	15 (22.1%)
**III/IV**	53 (77.9%)
**Pathology (WHO 2005)**	
WHO type 1 (SCC, keratinized)	24 (35.2%)
WHO type 2 (SCC, non-keratinized)	4 (5.9%)
WHO type 2.2 (Undifferentiated)	38 (55.9%)
Others	2 (3%)

### Radiation doses to the inner ear

For 41 patients who received conventional RT, dosimetric data were not available. For 27 patients who received IMRT, 54 ears were re-analyzed. Mean doses to the cochlea, inner ear and IAC were 51.02 Gy (range 25.09 - 75.54), 45.32 Gy (range 19.86-75.55) and 50.51 Gy (range 27.75-73.29), respectively.

### Chemotherapy

Chemotherapy was given to 97% of the patients (66/68 patients). Most of the patients received concurrent chemoradiotherapy followed by adjuvant chemotherapy. Sixty two patients received Cisplatin, while 4 patients received Carboplatin. The total accumulative doses of Cisplatin ranged from 120 mg to 980 mg (median dose 689 mg, mean dose 639 ± 233 mg). Carboplatin accumulative doses ranged from 200 mg to 2100 mg (median dose 980 mg, mean 988 ± 670 mg).

### Treatment outcomes

Median follow up time for all patients was 27.5 months (range 8-65 months). At the end of the study, 13 out of 68 patients were lost to follow up. The 2 year-progression free survival of this study group was 76.4% with a 2 year locoregional control of 88.5%.

### Audiological assessment and the incidences of post radiation SNHL

Pre RT audiograms demonstrated that 65.5% of the ears (88/134 ears) were normal or had mild BC hearing losses (16-25 dB) at 4 kHz. At PTA, 91% of the ears (122/134 ears) were normal or had mild hearing losses.

Post RT audiograms were performed at different follow up intervals. The median follow up time of audiological assessment for all 68 patients was 14 months (range 6-43 months). Median audiological follow up times for conventional RT and IMRT groups were 15 months (range 6-43 months) and 13 months (range 6-29 months), respectively. For total of 68 patients (134 ears), the incidence of SNHL at high frequency (4 kHz) was 52.9% (unilateral loss 13/68 patients, bilateral loss 23/68 patients). At PTA, the incidence of SNHL was 10.3% (unilateral loss 6/68 patients, bilateral loss 1/68 patients). For individual ear evaluation, the incidences of SNHL were 44% (59/134 ears) and 6% (8/134 ears) at 4 kHz and PTA, respectively.

### Factors associated with the incidences of SNHL

#### Radiation techniques

With conventional RT, the incidences of SNHL were 48.75% (39/80 ears) at 4 kHz and 5% (4/80 ears) at PTA, respectively. With IMRT, the incidences of SNHL were 37% (20/54 ears) at 4 kHz and 7.4% (4/54 ears) at PTA, respectively.

#### Radiation doses to the cochlea, inner ear, and IAC

Mean radiation doses to the cochlea, inner ear and IAC in this study were about 50 Gy, 45 Gy and 50 Gy, respectively. The authors then evaluated the incidences of SNHL based upon the mean radiation doses to each inner ear structure as shown in Table 2.

**Table 2:** The incidences of SNHL and the inner ear mean radiation dose (IMRT)

Mean radiation doses	Total 54 ears	SNHL at 4 Hz (ears)	SNHL at PTA (ears)
**Cochlea mean dose**			
≤ 50 Gy	24	6/24 (25%)	0/24 (0%)
> 50 Gy	30	14/30 (46.67%)	4/30 (13.3%)

**Inner ear mean dose**			
≤ 45 Gy	29	8/29 (27.59%)	0/29 (0%)
> 45 Gy	25	12/25 (48%)	4/25 (16%)

**IAC mean dose**			
≤ 50 Gy	31	8/31 (25.81%)	0/31 (0%)
> 50 Gy	23	12/23 (52.17%)	4/23 (17.4%)

**Figure 3:**
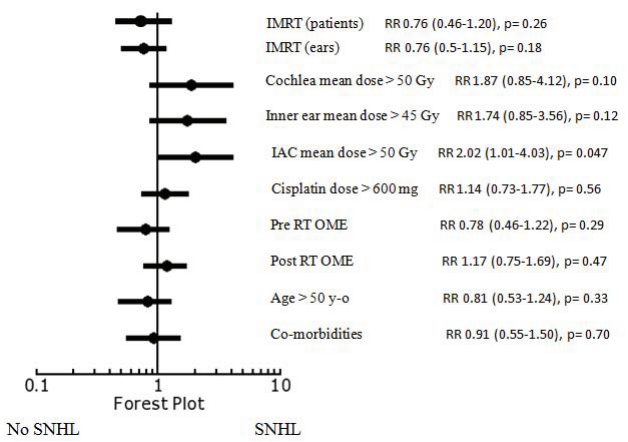
**Forest plot for relative risk of SNHL at 4 kHz**.

**Figure 4:**
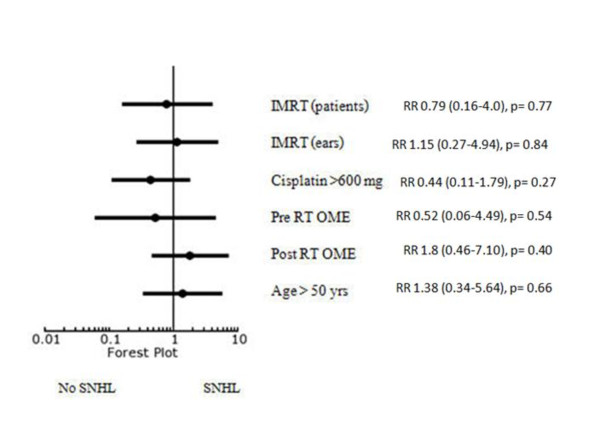
**Forest plot for relative risk of SNHL at PTA**.

On univariate analysis; IMRT, cochlea mean dose ≤ 50 Gy, inner ear mean doses ≤ 45 Gy and IAC mean dose ≤ 50 Gy appeared to have lower incidences of SNHL at high frequency (4 kHz). The other associated factors, including Cisplatin doses, OME, age and co-morbidities of the patients were not demonstrated to affect the incidence of SNHL (Figure 3). At PTA, there was no significant factor affecting the incidence of SNHL (Figure 4).

Based on the literatures reviewed, most studies reported that the incidences of SNHL were impacted by mean cochlea doses in the range of 45-50 Gy [4,5,7,11]. We therefore did not re-explore the data as quantitative continuous variables. Instead, we re-validated the known cut-off point starting at 50 Gy, which was actually the mean cochlea dose in our study. The lowermost cut-off level at 45 Gy was chosen for analysis [7]. The data showed that the RR for mean dose of > 45 Gy was 1.77 (95% CI 0.82-4.24) at 4 kHz, compared to dose ≤ 45 Gy. This analysis showed that the incidence of SNHL was not significantly changed when mean cut-off doses to the cochlea decreased from 50 to 45 Gy.

Based on the cochlea nerve tolerance of 54 Gy, we then explored the optimal radiation threshold to the IAC by creating a hypothesis with a cut-off point of 54 Gy. The data showed that the RR for a mean dose > 54 Gy was 2.25 (95% CI 1.14-4.17) compared to a mean dose ≤ 54 Gy at 4 Hz.

As radiation techniques had a potential effect on SNHL, we had analyzed the effect of IMRT on different variables with bivariate analysis. The bivariate analysis demonstrated that IMRT tended to decrease SNHL in younger patients (≤ 50 years old) or healthy patients without medical co-morbidities (DM and/or hypertension) (Table 3).

**Table 3:** Bivariate analysis (Effect of IMRT on different variables)

Factors	Relative risk	Test for homogeneity
**Age (years old)**		p = 0.40
> 50	1.00 (95% CI 0.49-2.10)	
≤ 50	0.68 (95% CI 0.39-1.18)	

**Cisplatin (total dose, mg)**		p = 0.57
> 600 mg	0.94 (95% CI 0.59-1.49)	
≤ 600 mg	0.70 (95% CI 0.28-1.73)	

**Co-morbidities**		p = 0.36
Yes	1.11 (95% CI 0.45-2.78)	
No	0.69 (95%CI 0.43-1.12)	

As NPC patients required Cisplatin chemotherapy to ensure the local and distant control, SNHL was potentially worse when combined with high radiation doses to the hearing structures. The authors then performed a bivariate analysis to evaluate the effects of Cisplatin on radiation dose levels for each inner ear structure. An accumulative Cisplatin dose of > 600 mg was used in this analysis since the mean Cisplatin dose delivered was about 600 mg in our study. The data demonstrated that in patients who received higher dose of Cisplatin (> 600 mg), the incidences of SNHL tended to be higher if they received mean radiation dose of > 50 Gy to the cochlea and > 45 Gy to the inner ear (Table 4).

**Table 4:** Bivariate analysis of Cisplatin effect on radiation dose levels.

Factors	Relative risk	Test for homogeneity
**Cochlea mean dose (Gy)**		p = 0.21
> 50	2.1 (95% CI 0.62-7.15)	
≤ 50	0.63 (95% CI 0.15-2.67)	

**Inner ear mean dose (Gy)**		p = 0.14
> 45	3.06 (95% CI 0.51-18.33)	
≤ 45	0.67 (95%CI 0.2-2.19)	

**IAC mean dose (Gy)**		p = 0.89
> 50	1.33 (95% CI 0.47-3.78)	
≤ 50	1.18 (95% CI 0.29-4.81)	

## Discussion

Radiation induced SNHL has been recognized as an important adverse effect which generally develops 6 to 24 months after radiation treatment and may progress to complete deafness [10,12]. The inner ear is the most susceptible organ for a durable long term SNHL. The etiologies of RT induced SNHL are vascular insufficiency, reduced number of capillaries, degeneration of endotheliocytes in vessels, loss of cells in the organ of Corti, atrophy and degeneration of the stria vascularis, and atrophy of the spiral ganglion cells and the cochlea nerve [13,14]. This damage is more prominent to the outer hair cells in the basal turn of the cochlea, which is responsible for transduction of higher frequency sound and a clinically significant SNHL at a higher frequency (>2 Hz) might occur.

The incidences of radiation induced SNHL were reported in the range of 0-65% with various radiation techniques (Table 5). Our study demonstrated that the incidences of SNHL were 44% (59/134 ears) at high frequency (4 kHz) and 6% (8/134 ears) at PTA for the whole population. Each study, however, was performed and evaluated with different criteria and follow up times.

**Table 5:** Criteria and radiation doses to the cochlea in correlation with the incidences of SNHL

Study	RT	Criteria	Doses to cochlea	Median follow up & SNHL (per ear)
Kwong et al[10]	Conv RT + chemo (227 ears)	>15 dB at each frequency	not defined	30 months 24.2%

Oh et al [19]	Conv RT + chemo (48 ears)	>15 dB at 4 kHz and PTA	mean inner ear dose 66.2 ± 6.2	1 year 29.2%

Ho et al [12]	Conv RT + chemo (526 ears)	>10 dB at 4 kHz and PTA	estimated 70-91 Gy, 2.5-3.5 Gy/F	4.5 years, 4 kHz 60% PTA 18%

Chan et al [5]	Conf RT vs Conf RT + chemo (170 ears)	>15 dB at 4 kHz	mean cochlea dose 33-71.7 Gy	24 months 33.3% vs 55% (Conf RT vs Conf RT+ chemo)

Chen et al.[4]	Conf RT+ chemo (44 ears)	>20 dB at one frequency >10 dB at two frequencies	28.4 - 70.0 Gy	29 months 57%

**Our study**	Conv RT +chemo vs IMRT+ chemo (134 ears, 68 patients)	>15 db loss at 4 kHz and PTA	Mean cochlea dose 25.09-75.54 Gy (IMRT)	14 months 4 kHz Conv 48.75% IMRT 37% PTA Conv 5% IMRT 7.4%

Conv RT = Conventional radiation therapy Conf RT = Conformal radiation therapy

The median follow up time (14 months) for audiological assessment in this study was rather shorter than the other studies. Nonetheless, radiation induced ototoxicity is typically evident at 6-12 months after completion of radiation therapy [4]. Transient SNHL might occur up to 41% of the patients as reported by Ho et al [12]. This study was not able to evaluate the transient hearing loss because of its retrospective design which was based upon different follow up times.

The hospital's policy is to routinely perform the audiological assessment for all NPC patients. However, a number of patients in our study did not complete the audiological tests. We recognized that the completeness of audiometric evaluation for every patient would be challenging in the absence of a prospective clinical trial. In this study, we compared the differences of pre and post RT audiograms rather than using the specific hearing threshold to justify the SNHL. We also excluded the patients who had only post RT audiograms. This should diminish the bias from patients who performed audiological exams because of having hearing impairment post RT.

The prior studies included only patients who completed the audiological assessment. This would potentially alter the incidence of SNHL in a certain number of patients who never performed the audiological exams. Also, the usage of contralateral ear as a baseline could create some inconsistency of the results [4,15,16]. Thus, we assumed that the results of our study would be adequate to report the incidence of SNHL although we understood the weakness of retrospective data.

With different radiation techniques, IMRT was found to have fewer incidence of SNHL when compared to the conventional RT (37% vs 48.75%). There was a trend to decrease the incidences of SNHL with IMRT in our study (RR of 0.76 with 95% CI 0.5-1.15, favouring IMRT). The former studies for NPC treatment had not directly compared the incidences of SNHL between conventional technique and conformal techniques. By indirect comparison among studies, the incidences of SNHL with the conformal techniques were not consistently lower than the conventional technique as shown in Table 5. The delineation of the normal structures and radiation dose constraint are very important in IMRT planning. IMRT potentially provides higher radiation doses to the cochlea than three dimensional conformal radiation technique, or even more than conventional RT, if the cochlea is not intentionally avoided [17].

In this study, we had delineated the inner ear into the cochlea and inner ear as there was some disagreement of cochlea delineation among the earlier studies [5,7]. Because of the tiny volume of the cochlea, target delineation is essential for dose volume analysis. Especially, its location is in the high dose gradient of the IMRT.

The results from our study demonstrated that the incidence of high frequency SNHL tended to be increased when mean dose delivered to the cochlea was > 50 Gy. Earlier studies suggested that the incidences of SNHL were increased with mean cochlea doses > 45-50 Gy [4,5,7,11]. However, our exploratory analysis showed that the incidence of SNHL was not significantly changed when mean cut-off doses to the cochlea decreased from 50 to 45 Gy. Therefore, our study suggested that the mean cochlea dose of 50 Gy should be reasonable since the excessive dose constraint to the cochlea would potentially compromise the nearby targets coverage.

Apart from the cochlea, the IAC should be concerned as the cochlea nerve traverses through the canal entering into the brainstem. The SNHL due to a retro-cochlea (cochlea nerve) damage may occur, although, this was relatively rare compared to the cochlea damage [18]. IMRT could deliver higher doses up to 66 Gy to the IAC if the IAC was not specified as the organ at risk [19]. This study demonstrated that the IAC mean doses > 50 Gy showed a trend to increase the incidence of high frequency (4 kHz) SNHL (RR 2.02 with 95% CI 0.99-4.13). IAC dose limitation was also crucial as the patients who developed SNHL from cochlea nerve damage would not obtain any benefits from using a hearing aid or even a cochlea implantation.

Our study revealed the lower incidence (10.3%) of low frequency SNHL (PTA). This concurred with the other series, as the high frequency (> 4 kHz) would be the earliest sign for damage at the outer hair cells in the basal turn of the cochlea [19].

Another coexisting factor for high frequency SNHL was a combination of Cisplatin chemotherapy with RT from a synergistic effect to the cochlea [4,5,15,20]. Some series reported that 600 mg/m^2 ^[21] or total dose of 1,050 mg [22] of Cisplatin increased the incidences of high frequency SNHL. As most of the patients in this study had locally advanced disease and received a combination of chemotherapy, the effect of Cisplatin to SNHL could not be evaluated directly. There was no apparent increase in the incidence of SNHL with a total accumulation dose of > 600 mg in this study. However, a further bivariate analysis revealed that dose limitation to the cochlea (< 50 Gy) and inner ear (< 45 Gy) would potentially protect SNHL in patients who received Cisplatin chemotherapy to an accumulative dose of > 600 mg.

For the other associated factors, Kwong et al reported the association of age, sex, and post RT serous otitis media as significant prognostic factors for persistent SNHL on multivariate analysis [10]. Nonetheless, this study could not demonstrate any relationship among age, evidence of otitis media, and/or medical co-morbidities with the incidence of SNHL.

Lastly, the inter-fraction setup uncertainties for very small structures are crucial. Radiation dose evaluation on the computer planning would be only the estimation of the actual dose delivered to the tiny inner ear during the radiation course.

## Conclusion

Apparently, radiation therapy produces relatively high incidences of high frequency SNHL. The severities of the damage are increased with higher radiation doses delivered to the inner ear structures. Mean radiation dose constraint of 50 Gy to the cochlea and IAC showed a trend to decrease the incidences of SNHL, especially in patients who received combination of Cisplatin chemotherapy. Normal structures delineation and radiation dose constraint with modern radiation techniques are crucial to diminish the long term SNHL and enhance the quality of life in addition to insuring survival from the cancer.

## Competing interests

The authors declare that they have no competing interests.

## Authors' contributions

JP designed the study, analysed the data and prepared the manuscript. AS carried out data collection, data analysis and helped to draft the manuscript. KT participated in the design of the study and performed the statistical analysis. PK participated in its design and hearing data evaluation. KT participated in hearing data evaluation. YC participated in its design and helped to draft the manuscript. PP participated in its design and helped to draft the manuscript. All authors read and approved the final manuscript.

## Authors' information

JP is an assistant professor in radiation oncology, focusing in head and neck cancer treatment. AS is a former radiation oncology resident. She is now a radiation oncologist at the regional cancer centre. KT is an instructor in radiation oncology, also a research facilitator in radiation oncology field. PK is an assistant professor in oto-rhino-laryngology, focusing in head and neck cancer surgery. KT is an instructor in oto-rhino-laryngology, specialized in otology. YC is an associated professor in radiation oncology. PP is a professor in radiation oncology, and a head of radiation oncology division.
